# “We Are from Nowhere”: A Qualitative Assessment of the Impact of Collective Trauma from the Perspective of Resettled Bhutanese Refugees

**DOI:** 10.1089/heq.2020.0116

**Published:** 2021-10-28

**Authors:** Manisha Salinas, Juan L. Salinas

**Affiliations:** ^1^ Center for Health Equity and Community Engagement Research, Mayo Clinic, Jacksonville, Florida, USA.; ^2^ Department of Sociology, Anthropology, and Social Work, University of North Florida, Jacksonville, Florida, USA.

**Keywords:** Bhutanese refugees, collective trauma, displacement, mental health, integration

## Abstract

**Background:** Limited research has explored mental health concerns and collective trauma experienced by Bhutanese refugees due to their displacement from Bhutan, refugee camp life in Nepal, and resettlement to U.S. society.

**Purpose:** To understand how collective trauma experienced by Bhutanese refugees influences the process of resettlement and integration into U.S. society to better address mental health concerns from the community.

**Methods:** Qualitative data were collected through four focus groups (*N*=40) with Bhutanese refugee women in central Massachusetts from June to November of 2016 to discuss refugee resettlement experiences and mental health concerns.

**Findings:** Bhutanese refugees shared insights on their resettlement experiences where several broader themes emerged, including *historical collective trauma*, *closed-door culture*, and *processing mental health stigma*. The displacement from Bhutan, hardships in Nepal refugee camps, and isolation in U.S. society led to a collective trauma among the community. Participants described America as having a closed-door culture that limits their integration into society, causing unique challenges based on their context of integration. The collective trauma also poses challenges toward processing mental health stigma, yet community building offers insights on how Bhutanese refugees can address these issues in collective spaces.

**Conclusions:** The historical collective trauma must be considered when working with Bhutanese refugees to understand the context of their resettlement to address mental health concerns.

## Introduction

Over 26 million refugees have been forcibly displaced from their home countries due to violent conditions, political conflict, and war.^1^ Even after their displacement, temporary refugee camps continue to be unsafe, leading to further resettlement to other countries.^2^ The trauma of relocating multiple times is destabilizing as it creates substantial uncertainty, a rupture in community embeddedness, and conditions leading to isolation from continuous frustrations with inadequate social institutions.^3,4^ Adapting to a new way of life places a burden on refugees already adjusting to the challenges of displacement; which include social isolation, marginalization, discrimination, and lack of access to social, cultural, and economic resources.^5^ This has become a major public health concern, as the multiple stressors faced post-resettlement can be mitigated by effective health promotional efforts, such as health education on coping strategies, community building and organizing, and increasing access to appropriate health services.^5^

To improve the conditions of these groups, it is important to gain an understanding on the collective trauma of refugee communities, which stems from decades of oppression and isolation with intergenerational impact and detrimental mental health effects of the entire group through shared traumatic history.^6^ To make progress toward health equity, it is necessary to acknowledge the stories of the community and incorporate their perspectives on disparities they encounter. By taking into account the specific cultural considerations of the community, is it possible to increase the utilization of health resources, reduce stigma of sensitive health concerns, and take a step closer toward collective healing.^7^

The focus of this study is the Nepali-origin Bhutanese, who represent over 100,000 individuals relocated in the resettlement process, with at least 96,000 being resettled into the United States by 2019.^8^ Multiple relocation and removal of this communities from various regions have forced a lifetime of readjustment-related stressors when adapting to new cultures and lifestyles.^4,9,10^ Social isolation and lack of availability of culturally sensitive health services have shown to be risk factors for health disparities and unequal access to care among these groups.^9–12^ Similar to other refugee groups, this can make them increasingly at risk for poor physical and mental health outcomes.^13,14^

The mental health status of Bhutanese refugees has been a rapidly growing area of concern, as there have been high rates of suicide and suicidal ideation among this group post-resettlement.^8,9,11,15^ Although mental health of resettled Bhutanese refugees remains an ongoing priority, limited studies have explored the stories of Bhutanese refugees in-depth to address challenges to health equity and community-identified strategies to mitigate the impacts of collectively traumatic experiences of this group.^16^ This study aims to explore the perceptions and challenges experienced by a resettled Bhutanese refugee community to reveal the impact of displacement on the mental health of this group based on their specific social and cultural context. Findings from this study can serve to inform culturally responsive approaches to reducing mental health burdens and developing relevant focused programs for the community.

## Methods

### Study site and recruitment

This research was conducted in Worcester, MA, a city in central Massachusetts with a population of over 185,000.^17^ Worcester is home to some of the highest numbers of resettled refugees in the state.^18^ There are a number of organizations found in this city to assist refugees in their transition to a new setting, however, refugees are often left to independently navigate their new lives, and those with limited English proficiency or other barriers face many challenges and difficulties, impacting their mental and emotional health.^19^ A resource guide for Worcester refugees points to the minimal visibility of services and the challenges in connecting refugees to assistance after their initial 90-day resettlement period.^19^ The Bhutanese refugee community is among the largest in Worcester, and have established local resources, religious centers, and cultural social spaces as they settled into the city.^17^ Many Bhutanese refugees live in multi-family homes within close distance of one another. The close-knit ties of the community allowed for recruitment through trusted community leaders using snowball sampling and by word of mouth to spread information about the study. For those who were interested to participate in the study, convenient times and locations were secured, with three focus groups conducted within the homes of participants and one at a local community temple. Eligibility criteria, included adult women of Nepali ethnicity identifying as Bhutanese refugees. The study participants were compensated with a small gift card for their time. This study was approved by the Institutional Review Board through the Division of Research at Texas A&M University.

### Data collection

Data collection occurred through focus group discussions that were used to explore the experiences and perspectives of the Bhutanese refugees and the impact of displacement and resettlement process on their mental health concerns. Focus groups support the collective sharing of attitudes toward health issues and community perceptions on social issues, as well as a space for mutual support.^20^ There were a total of forty participants across four focus groups taking place from June to November 2016. The first author is fluent in Nepali and familiar with Nepalese culture and served as the focus group moderator based on her training in qualitative methodology and focus group facilitation. Participants took turns addressing their concerns and they were encouraged to share their thoughts. These efforts were made to be inclusive of various perspectives to create a comfortable space to share experiences.^21^ At the start of the discussion, a community leader introduced the study, and the remainder of the discussion was led by the primary author in the Nepali language. Participants gave verbal informed consent for audio recording and all remained for the duration of the focus group discussion. A semistructured guide found in Table 1 was modified for cultural relevancy based on community leaders' feedback before the study, and each focus group lasted up to 2 h. After the focus group discussion, a demographic questionnaire was distributed to participants. Table 2 displays the aggregated sociodemographic data per focus group. Overall, the average age was 48 and length of time in the United States was 3 years. The majority of the participants had <3 years of formal education, lived in refugee camps for 19 years before resettlement, and rated their health as fair on a 5-point scale.

**Table 1.** Sample Questions Focusing on Mental Health and Resettlement Processes for Focus Group Discussion with Bhutanese Refugees

Focus group questionnaire
1. How is getting health services here different than in Nepal/refugee camp/Bhutan?
2. Where do you, or people you know, go in Worcester for help or advice for health issues?
3. How has the process of adjusting to a new life and/or culture gone for you? What about others that you know? Do you think others are having similar or different experiences adjusting?
4. How is mental health seen or addressed in your community?
5. What are the most important health concerns for you and/or your family?
6. How is getting health services here different than in Nepal/refugee camp/Bhutan?
7. Where do you, or people you know, go in locally for help or advice for health issues?
8. Are there any health services that you feel Bhutanese refugees here need but do not have?
9. Mental health can be a concern for refugees when dealing with the stress and anxiety of change to adjusting to a new life away from home. How is mental health seen or addressed in your culture?
10. Are there any other things important to you about Bhutanese refugee health that we haven't asked you about?

**Table 2.** Focus Group Participant Characteristics (Averages) Based on Demographic Information Obtained Through Questionnaires (*N*=40)

Focus group	Total participants	Average age	Years in refugee camp, years	Time in United States/Worcester, years	Years of formal education	Self-reported English proficiency	Self-reported health status
1	10	47	21	3	3	Fluent (1); Some (2); Little (3); None (4)	Excellent (1); Very Good (0); Good (3); Fair (4); Poor (2)
2	10	53	17	4	1	Fluent (1); Some (2); Little (2); None (5)	Excellent (0); Very Good (0); Good (3); Fair (6); Poor (1)
3	12	45	18	5	2	Fluent (0); Some (4); Little (3); None (5)	Excellent (3); Very Good (2); Good (1); Fair (4); Poor (2)
4	8	50	18	5	3	Fluent (1); Some (1); Little (3); None (3)	Excellent (0); Very Good (1); Good (2); Fair (4); Poor (1)

### Data analysis

Audiorecorded focus group discussions were transcribed verbatim by the first author, with pseudonyms used throughout transcriptions and final reports. After the transcripts were transcribed, they were back translated from Nepali to English and reviewed by a certified bilingual health care interpreter. Data were drawn from focus group transcripts, memos, and field notes from a larger study and qualitatively analyzed using ATLAS.ti (v7) software.

Data were thematically coded and categorized to interpret meanings based on shared patterns until thematic saturation was reached.^22^ Thematic concepts emerged during the coding process, which were organized based on commonalities of codes.^23^ These codes were conceptually categorized through constant comparison and analyzed based on patterns in the data.^22,23^ Throughout the analysis process, the authors discussed the content of the quotes and participants' meanings of responses. Discrepancies were discussed among the authors until consensus was reached on the categorization of themes. The emerging themes were conceptualized, defined, and reported with the corresponding subthemes and supporting quotations described in Table 3.^22^

**Table 3.** Major Themes, Supplementing Subthemes, and Example Quotes from Participants in Focus Group Discussions

Major themes	Description	Subthemes	Example quotes
Historical collective trauma	Development of collective trauma through expulsion from Bhutan and enduring refugee camps within Nepali society	*Surviving ethnic cleansing* We are from nowhere Despair adjusting to refugee camps	We did not even get to get our clothes that were drying outside after just being washed. The clothing line was full of clothes. We had the lights on and left the door open, we had big containers of rice and food we just left it all like that. We left the crops that we were about to harvest, seeds for next season, partially harvested rice, beans; we had oranges, other fruits. We left with everything as it was; we had to leave with just the clothes on our back. What can you do? We survived from that, and we are still surviving. (Indra, Focus Group 1) We thought we were from Bhutan, and we stayed there thinking that. But they said ‘No, you are not. You are from Nepal. Your language and our language are not the same.’ and they kicked us out. Then, in Nepal, we went there thinking it was our place, our home. But in Nepal, they said, ‘You are not *our* kind of Nepali. You are from Bhutan.’ They did not give us citizenship from [Nepal], and they kicked us out in [Bhutan]. We are from nowhere. (Saru, Focus Group 2) People in our community left their homes and where they are from, and they do get depression. They still face hardship here, and they do not get help. Our neighbor, he took his life last year. He may have thought he didn't have another choice. We have heard of many stories like that in other Bhutanese refugee communities also. (Tara, Focus Group 2)
Closed-door culture	The perception of an individualistic culture after transitioning from a collectivist community	*Collectivist to individualistic society* *Feeling invisible*	When I first came here, we thought there were no others like us, right? There was no way of knowing who people were. People here just go inside and close the door, and you have no idea who is back there. […] People can get depressed just keeping these [feelings, thoughts] inside and not sharing. Who else can we talk to though? We can't understand people outside. And if you try to see your neighbor here, the doors stay closed. (Sita, Focus Group 2) Many people would be there in the village, if only one person was ill. Here, it is not like that, and people are alone if they do not have others in their community. (Tara, Focus Group 2) For me sometimes it feels just like I am choking. And why? I do not have education, I cannot read and write, I do not know how to speak [English]…I see people walking around but I cannot say hi or I do not know what to say. I want to talk to them, I want say hi. But they do not understand what I say and I do not understand what they say. And so I am walking around like a dumb person not knowing what is happening. (Nina, Focus Group 1)
Processing mental health stigma	The challenges and strategies to address the mental health in the community	*Social repercussions of labeling* Safe space for mental health discussions	Even if someone has a mental health problem in the community, they do not want to come forward and say it. Even if they know there are mental health services, they don't want to use it because in our community people may say “oh, he's *crazy*?” So they do not come out about that, and they will not talk about it. (Puja, Focus Group 2) People will fear that this person will tell another one, and that it will be like in the camps. So they fear that and so they don't open up. And they still hide the problem. Even if it is serious, they don't want to tell to the family members thinking it hurts them and they feel bad and they don't want to let them know. They also feel others should not know in our society. (Jyoti, Focus Group 1) Having people to trust, it is important. There are many things that are uncomfortable to share…So making a time to get together, to talk with each other about specific things would help. And we should make sure people do not gossip about others' problems, by saying that everything we talk about stays in this room, so that there is a place they can trust to talk about their problems. (Mina, Focus Group 3)

## Findings

Three major themes emerged from participants' focus groups; (1) *historical collective trauma*, (2) *closed-door culture*, and (3) *processing mental health stigma*. *Historical collective* trauma refers to the trauma the Bhutanese refugees experienced through expulsion from Bhutan as well as enduring the refugee camp life in Nepal. *Closed-door culture* refers to how Bhutanese refugees struggle with the transition from a collectivist culture into an individualistic culture in U.S. society. *Processing mental health stigma* are the reflections on the challenges and strategies Bhutanese refugees use to address mental health in the community. These major themes are mentioned throughout the focus groups by participants as they shared their concerns about their refugee status and mental wellbeing. Table 3 displays quotes for each of these themes that reflect the major findings from the study that emerged during the analysis. While the focus group settings provide a variety of perspectives, we aimed to describe general patterns that emerged in conversations with participants.

### Historical collective trauma

#### Surviving ethnic cleansing

The Bhutanese refugee community carries historical collective trauma based on surviving ethnic cleansing, expulsion from Bhutan, and adjusting to refugee camp life in Nepal.^24^ The painful nature of expulsion and forced resettlement in refugee camps have significant implications for the lives of Bhutanese refugees. These historical traumas influence the perceptions and interactions Bhutanese refugees have as they later experience integration into U.S. society.

Participants shared their hardships as they faced political conflict and ethnic cleansing in Bhutan that resulted in their expulsion and later difficulties as refugees in Nepal. In Table 3, a quote by Indra (Focus Group 1) explains the experience of being rushed out of Bhutan, leaving behind their property and all of their belongings. These circumstances were highly traumatic, as these Nepali/Bhutanese refugees faced a policy of ethnic cleansing when the Bhutan government implemented the campaign of “One Nation, One People” to reinforce the Buddhist identity of Bhutan and remove ethnic minorities.^24^ The Nepali/Bhutanese, despite living several generations in Bhutan, were stripped of their rights as citizens and are now considered as having an undocumented status for not having legally recognized paperwork by the Bhutanese government.

#### We are from nowhere

The participants shared how this displacement from losing their homeland was a significant impairment to their identity and sense of belonging to any place. Saru (Focus Group 2) explains that they were frequently told they did not belong in Bhutan leading to their expulsion and that they were not the right kind of Nepali to be in Nepal, concluding they “were from nowhere,” without a sense of belonging to any nation. This displacement and expulsion included forced relocation to Nepali refugee camps where they were still considered outsiders because of their distinct Bhutanese/Nepali dialect and prior Bhutanese residency. This exclusion has ranged in the way they faced institutional discrimination and social hardships adjusting to refugee camp life.

#### Despair adjusting to refugee camps

Facing ethnic cleansing in Bhutan they were forced into relocation in Nepal, but although they were physically safer from ethnic persecution, they lived in these temporary Nepali refugee camps for decades with their own social problems. The pain of losing home leads to a common theme referred to as despair adjusting to refugee camps among the participants. Tara (Focus Group 2) spoke about the hardships faced by the refugees that had to collectively leave their homes they had held for generations only to find themselves adjusting to a new collective struggle in refugee camps without adequate support. Tara also shares how suffering runs deep in the community, that suicide is common, and that there continues to be a lack of resources to address this collective trauma and depression faced by Bhutanese refugees. The hardships of political persecution, environmental harms, and poverty in refugee camps are internalized forms of trauma that were not adequately addressed by existing resources. This context must be understood when considering the Bhutanese refugee arrival to the United States.

### Closed-door culture in the United States

#### Collectivist to individualistic society

As Bhutanese refugees began to arrive in the United States, their collective trauma continued to significantly impact their experiences and integration into U.S. society. Sita (Focus Group 2) described U.S. culture as a “closed door” both in a physical and symbolic sense, referring to the way they noticed Americans keeping their doors closed and maintaining a distance from their neighbors. This was in stark contrast to the collectivist society they were accustomed to, as described by Tara (Focus Group 4), who discusses the openness and communal support provided by an entire village in times of need. The transition from an open society to a closed one is especially difficult for those that first arrived, as they described a significant feeling of culture shock and being left to navigate a complex individualistic society on their own. The closed doors of the neighbors in their lives post-resettlement symbolized feeling shut out of society and participants described feeling forced to deal with their experiences alone. Integration into U.S. society became an isolating experience which had to be managed without outside assistance.

#### Feeling invisible

Furthermore, many refugees faced additional barriers, such as older age, limited English proficiency, and economic insecurity, and felt a significant disconnect when trying to communicate. In Table 3, Nina (Focus Group 3) describes feeling cut off from the ability to meaningfully interact with others in U.S. society. She uses the analogy of choking or suffocating to illustrate her inability to speak or interact with the rest of society because of her limited English ability and lack of education. This captures the social isolation Bhutanese refugees experience that can lead to implications for their physical and mental wellbeing.

### Processing mental health stigma

#### Social repercussions of labeling

The experiences of collective trauma, social isolation, and transitioning to a new culture placed a significant burden on the mental health of the Bhutanese refugees. Although there is a strong sense of community among them, mental health is still viewed as a private, individual problem and there continues to be a stigma in seeking treatment. Puja (Focus Group 2) discusses the fear of being labeled as “crazy” by other Bhutanese refugees for seeking mental health care.

Participants shared that refugees are not accessing mental health services because of the social repercussions of being labeled or “coming out” as having mental health problems. This often leads to keeping those mental health problems hidden or suppressed during conversations with doctors, family, and community members.

Another participant, Jyoti (Focus Group 1) discusses that even serious mental health issues are not brought up to family members to keep the burden to themselves. There is a fear of being ostracized or bringing shame to themselves and their family, leading many to suffer alone. The participant shared that this pattern was seen in the refugee camps in Nepal, in which community members with mental health problems would feel stigmatized by others in the camps. This stigma carries over into their communities in U.S. society and perpetuated through the underutilization of services particularly for mental health problems.

#### Safe space for mental health discussions

Although many participants acknowledge the issues of stigma in the community, the focus group setting became a place to share similar stories of coping and supporting one another throughout the duration of the discussions. In this safe space, Bhutanese refugees acknowledged the collective trauma experienced from displacement in Bhutan, relocation from Nepal, and resettlement in the United States, addressing the role of stigma throughout these social changes. Bringing mental health discussions out into the open helped identify issues that were impacting the community, and participants crafted potential solutions to address these problems. For example, toward the end of the focus group discussion, Mina (Focus Group 3) highlighted the benefit of having a safe, shared space for community members to voice their concerns to express needs, learn from one another, and create or strengthen bonds to build social support networks. Her words encouraged others to discuss committing to future group discussions to share stories and help each other with problems being faced.

## Discussion

The findings from this study explore the challenges of collective trauma faced by Bhutanese refugees through their lives. Their initial survival in the expulsion from Bhutan and subsequent hardships in the refugee camps resulted in historical trauma, felt collectively and with sense of loss of identity and security. Their resettlement in U.S. society presented continued feelings of unbelonging and invisibility. Participants described their physical, cultural, and social isolation from their neighbors and the difficulties in adapting to an individualistic society after being raised in a collectivist community. This increased their mental health burdens, which is in line with previous research demonstrating the adverse psychological impact of low social integration after the resettlement of traumatized refugees.^25,26^ Adjusting to the social structure of U.S. society is a long and arduous learning process, but having established social networks and high levels of social integration has been shown to significantly improve quality of life.^27^ In the Bhutanese community, part of this isolation was also the struggle of managing mental health problems alone.

Mental health is a sensitive health topic in Bhutanese refugee culture despite the close ties of the community. The unique conditions of refugee migration involve separation of families, fleeing violence or war, and the challenges of post-migration stress in U.S. society leads to increased risk of mental health issues.^8,28^ This collective trauma contributes to the high rates of depression, anxiety, post-traumatic stress disorder, and suicide or suicidal ideation.^4,8,9,11,28^

Participants often spoke about managing mental health problems while navigating the social repercussions of stigma. The fear of being ostracized or shamed in an environment, which is already overwhelming and complex, can contribute to many problems being kept hidden. The suicide rate of the Bhutanese refugee community has been growing at an alarming rate since their arrival.^8^ Therefore, the specific cultural considerations reported by these groups must be considered when addressing stigmatized health issues.

Although the initial conversations on mental health issues can be challenging, there is value to providing a trusted space for addressing these mental health concerns for community building and strengthening the social connectedness among vulnerable groups. The participants in this study shared experiences of trauma and isolation and their involvement in the focus groups encouraged them to discuss strategies on alleviating these burdens. The conversations provided a structure for community-led discussion groups in safe spaces for future dialogs on sensitive health topics.

Furthermore, there is a need to bring resettlement agencies, nonprofit services, and community organizations into the conversation with regard to refugee mental health. Addressing challenges and barriers at multiple levels, requires interorganizational collaboration and ongoing support, particularly since the impact of collective trauma and mental health issues happen over time. While there has been progress in addressing the needs of refugee as they initially settle into the United States, there needs to be a long-term commitment to the refugee community. This involves raising awareness of existing services, partnering with community members on addressing mental health needs, and collaboratively constructing solutions to improve responsiveness to mental health concerns.

This study demonstrated the challenges to collective trauma faced by Bhutanese refugees in a specific subpopulation. The findings may not be applicable to all the Bhutanese refugee community; although each struggle is unique, there are some patterns that have emerged from this study. The historical collective trauma was demonstrated by the experiences the participants had being displaced multiple times and feeling that they did not have a home or identity in their society. The difficulty in adapting to a radically different culture and ongoing stigmatized mental health concerns were acknowledged through their stories with each other. However, the strength and the resilience of the community was also demonstrated in their conversations to support one another and recommendations for increasing community support.

## Conclusion

Health care programs should consider refugee's historical collective trauma and the challenges this causes toward adjusting to a new language, culture, and community when integrating into U.S. society. Bhutanese refugees have a complex history of trauma, involving displacement and relocation from their countries of origin and transition. The resettlement process brings further challenges of new cultural adjustments that disrupt existing coping mechanisms with interacting with society. Researchers and program planners would benefit to spend time gaining an understanding of refugees' collective trauma and cultural norms. This could provide effective health outreach, education, and raise awareness of mental illnesses and social issues in the community. Understanding community concerns can help with reducing stigma and normalizing mental health issues to promote the wellbeing of individuals and their communities.

## Health Equity Implications

Refugees have complex histories, and a one-size-fits-all approach must be reconsidered to account for the context of refugees' collective trauma and cultural norms. The Bhutanese refugee migration experience is unique and follows collective trauma developed from ethnic cleansing in Bhutan, to hardships in Nepal refugee camps, to cultural barriers to adjust to the U.S. society. These experiences should be integrated into efforts when assisting refugees, developing appropriate community resources, and encouraging discussions to address mental health issues stemming from collective trauma. Furthermore, the focus group approach used in this study has a great potential for further community gathering, as participants shared the need for more discussions on sensitive health topics in safe communal spaces. Encouraging Bhutanese refugees to share their experiences brings opportunities to minimize stigma and spread an awareness of shared collective traumas to encourage mental health care discussions and potential treatments. There is an opportunity for significant changes to address mental health equity concerns in the Bhutanese refugee community as they face continual adjustments to life in U.S. society.
